# Descemet Stripping Endothelial Keratoplasty in a Patient with Keratoglobus and Chronic Hydrops Secondary to a Spontaneous Descemet Membrane Tear

**DOI:** 10.1155/2013/697403

**Published:** 2013-04-27

**Authors:** Anton M. Kolomeyer, David S. Chu

**Affiliations:** ^1^The Institute of Ophthalmology and Visual Science, New Jersey Medical School, University of Medicine and Dentistry of New Jersey, Newark, NJ 07103, USA; ^2^Metropolitan Eye Research and Surgery Institute, 540 Bergen Boulevard, Suite D, Palisades Park, NJ 07650, USA

## Abstract

*Purpose*. To report the use of Descemet stripping endothelial keratoplasty (DSEK) in a patient with keratoglobus and chronic hydrops. *Case Report*. We describe a case of a 28-year-old man with bilateral keratoglobus and chronic hydrops in the right eye secondary to spontaneous Descemet membrane tear. The patient presented with finger counting (CF) vision, itching, foreign body sensation, and severe photophobia in the right eye. Peripheral corneal thinning with central corneal protrusion and Descemet membrane tear spanning from 4 to 7 o'clock was noted on slit lamp examination. The right eye cornea was 15 mm in the horizontal diameter. After a 5.5-month loss to follow-up, the patient presented with discomfort, photophobia, decreasing vision, and tearing in the right eye. Vision was 20/60 with pinhole. 360-degree peripheral corneal ectasia with mild neovascularization and hydrops was present. Over the next few months, the patient complained of photophobia and intermittent eye pain. His vision deteriorated to CF, he developed corneal scarring with bullae, and a DSEK was performed. Eight months postoperatively, best-corrected vision improved to 20/30, cornea was clear, and the DSEK graft was stable. *Conclusions*. Nonresolving hydrops secondary to Descemet membrane tear in a patient with keratoglobus may result in permanent endothelial cell damage and scar formation. This may be successfully treated with DSEK.

## 1. Introduction

Keratoglobus is an idiopathic disorder with 360-degree peripheral corneal ectasia resulting in central corneal protrusion [1]. It is associated with ocular conditions such as orbital pseudotumor, vernal keratoconjunctivitis, chronic marginal blepharitis, and glaucoma after penetrating keratoplasty surgery; congenital conditions including Leber congenital amaurosis and blue sclera syndrome; and several connective tissue disorders, for example, Ehlers-Danlos syndrome, Marfan syndrome, and Rubinstein-Taybi syndrome [2–6]. Visual impairment in patients with keratoglobus can be profound, and may occur secondarily to corneal scarring and rupture (due to severe corneal ectasia), irregular astigmatism, and extreme myopia.

Hydrops develops due to breaks in the Descemet membrane followed by aqueous infiltration of the stroma and the epithelium in up to 91% of keratoglobus eyes, and can result in corneal scar formation in severe cases [7]. Other reported serious side effects include corneal perforation, microbial keratitis, and glaucoma [8, 9]. Factors predisposing to the development of hydrops include younger age, male gender, advanced corneal ectasia, and severe allergic eye disease [10]. Up to 60% of patients with hydrops may require a penetrating keratoplasty (PK) to achieve good visual outcome.

In the following, we describe a case of a 28-year-old man with keratoglobus and chronic hydrops secondary to spontaneous Descemet membrane tear who underwent a Descemet stripping endothelial keratoplasty (DSEK).

## 2. Case Report

A 28-year-old man presented to our clinic complaining of itching, foreign body sensation, and severe photophobia in the right eye for one week. Visual acuity (VA) with correction was finger counting (CF) and 20/40 in the right and left eyes, respectively. Intraocular pressure (IOP) was 11 mm Hg in the right eye and 18 mm Hg in the left eye. 360-degree peripheral corneal thinning with central corneal protrusion was observed bilaterally. The right eye had 1+ conjunctival injection, 3+ corneal edema, a Descemet membrane tear from 4–7 o'clock, and a 15-mm cornea in the horizontal diameter. Pentacam (OCULUS USA, Lynnwood, WA) of the left eye showed a 478 *μ*m central cornea, a 323 *μ*m peripheral cornea, and a 50.9 D K-max. Imaging of the right eye was not obtained due to an irregular and hazy cornea. The patient was diagnosed with bilateral keratoglobus complicated by hydrops in the right eye. He was prescribed prednisolone acetate 1% and cyclopentolate eye drops four times a day in the right eye.

After a 5.5-month loss to follow-up, he presented with discomfort, photophobia, decreasing vision, and tearing in the right eye for several months. VA was 20/150 with correction and 20/60 with pinhole in the right eye and 20/25 with correction in the left eye. The patient was prescribed oral prednisone 60 mg/day and prednisolone acetate 1% eye drops three times a day in the right eye. Mild corneal neovascularization and hydrops were noted on biomicroscopic examination of the right eye. VA, IOP, and clinical examination of the left eye were stable throughout the study period. All information presented henceforth relates to the right eye unless otherwise specified.

Ten days later, the patient complained of pain, irritation, and tearing. Vision decreased to 20/200 with no pinhole improvement. Conjunctival injection with corneal bullae was noted. A 14-mm bandage contact lens was placed, and Vigamox therapy four times a day and prednisone taper were initiated. A month later, the patient presented with photophobia and intermittent eye pain. VA was CF at six feet with correction and 20/40 with pinhole, IOP was normal, and a corneal scar with bullae was noted. A decision was made to perform a DSEK after a thorough discussion with the patient.

Conjunctival peritomies were created at 3, 6, and 10 o'clock positions, while paracentesis sites were formed at 3 and 6 o'clock. An anterior chamber (A/C) maintainer was placed at 6 o'clock. A scleral tunnel was created at 10 o'clock using a 2.75 mm blade to enter the A/C. Due to the extremely ectatic nature of the patient's cornea, the A/C was very unstable even with the maintainer in place. The crystalline lens was observed to move forward and contact the blade, which resulted in a small but definite rent in the anterior capsule. At this point, intraocular epinephrine was given to dilate the pupil, and viscoelastic solution was injected into the A/C for stability. Using a hook type instrument, the status of Descemet membrane was explored. A tear and a detachment was found spanning approximately from 4 to 7 o'clock along the peripheral cornea and extending into the central cornea. An anterior capsulotomy was completed and lens material was removed with phacoemulsification, after which stripping of the Descemet membrane was accomplished. Viscoelastic material was removed and a 9-mm corneal tissue graft was inserted using a glide and forceps. The wounds required several sutures each to close and the A/C was filled with air to maintain the graft in place.

**Figure 1:**
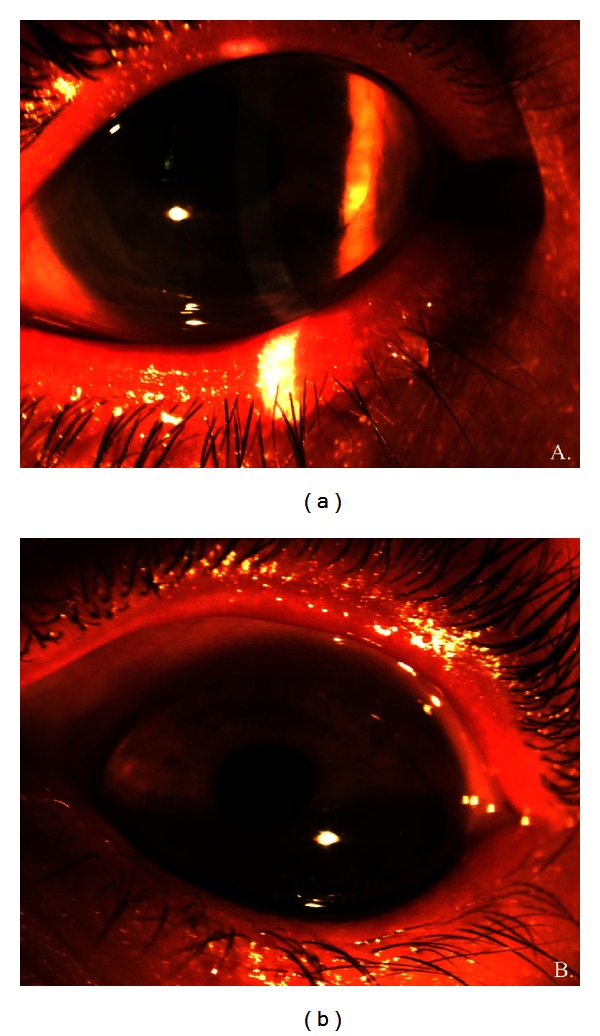
External photographs of the right (a) and left (b) eyes eight months after DSEK. (a) A 9 mm in diameter clear graft is evident. The graft appears small in an eye with a 15 mm cornea in diameter. (b) Diffuse ecstatic cornea is seen. The patient with keratoglobus is looking down slightly to show extreme corneal protuberance.

**Figure 2:**
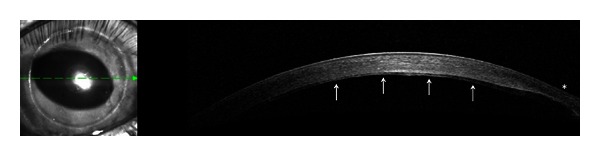
Anterior segment OCT of the right eye showing a stable DSEK graft eight months after placement (white arrows). Extreme peripheral corneal ectasia is evident (white star).

**Figure 3:**
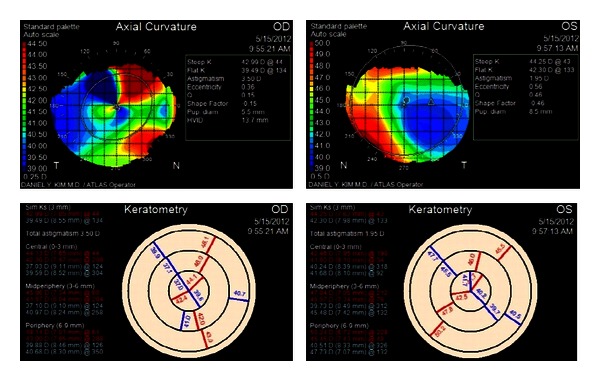
Axial curvature and keratometry measurements of both eyes eight months after DSEK graft placement in the right eye. Significant corneal irregularity is seen on the keratometric map in both eyes.

On postoperative day 1, the patient complained of photophobia and VA was hand motions (HM). DSEK graft was stable and 50% air in the A/C was noted. By postoperative week 1, the patient developed discomfort and tearing in addition to photophobia. Vision remained at HM, IOP was 17 mm Hg, and hydrops with a well-formed A/C was observed. A b scan was normal. By postoperative month 1, the VA improved to 20/200 uncorrected and 20/40 with pinhole. The cornea showed resolving hydrops. Two months after surgery, the patient complained of blurry vision and photophobia. Despite continued corneal clearing on biomicroscopic examination, VA decreased to 20/400 with correction and 20/50 with pinhole. By eight months after surgery, the patient reported significant improvement in photophobia, vision, and tearing as compared to the symptoms prior to DSEK placement (Figure 1). Best-corrected VA was 20/30 with a +3.50 sphere. IOP was normal, cornea was clear, and DSEK graft was stable (Figure 2). The keratometric map of both eyes is shown in Figure 3.

## 3. Discussion

Treatment options for managing patients with keratoglobus are limited and present a challenge due to a large thinned cornea. Possible treatment modalities include spectacle correction (in patients with clear corneas), hydrogel lenses (although contact lenses are not commonly used due to abnormal corneal topography and increased susceptibility to corneal rupture following minimal trauma), large-diameter or eccentric PK, large-diameter inlay lamellar keratoplasty (LK), tectonic LK followed by a PK, central LK with peripheral intralamellar tuck, deep anterior LK with big bubble technique, limbus-to-limbus epikeratoplasty (EK), limbus-to-limbus EK followed by a PK (in patients with a central corneal scar), and peripheral suturing of a corneoscleral ring graft [11–19]. The overall visual outcomes in keratoglobus patients are not optimal. 

Grewal et al. reported on medical management of acute hydrops complicating 19 eyes with keratoconus, two eyes with pellucid marginal degeneration (PMD), and one eye with keratoglobus [20]. When used singly or in combination, antibiotics, aqueous suppressants, cycloplegics, hypertonics, and/or steroids did not differ significantly in their effect on final visual outcome. In six patients with available pre-hydrops VA, post-hydrops VA remained the same or improved in five (83%) patients. Therefore, in cases of hydrops, although medical management should be considered initially, it is not likely to be sufficient. 

Few reports have addressed nonmedical management of hydrops complicating corneal ectasias (especially keratoglobus). Kaushal et al. used 0.3 mL of intracameral isoexpansile C_3_F_8_ gas to treat acute hydrops in a patient with PMD and hydrops [21]. They found significant improvement in VA and closure of an intrastromal cleft two weeks after gas injection without a rise in IOP or cataract formation. Kiire and Srinivasan used 1.0 mL of intracameral isoexpansile C_2_F_6_ gas to manage bilateral acute hydrops in a patient with bilateral keratoglobus [22]. At four weeks after injection, there was a marked improvement in vision, no photophobia, and hydrops was completely resolved bilaterally. However, the patient developed Urrets-Zavalia syndrome in the right eye. In a retrospective, comparative interventional case series of 152 eyes of 139 patients with corneal ectasias (72.4% keratoconus, 19.7% PMD, and 7.9% keratoglobus), Basu et al. showed that injection of 0.1 mL of intracameral isoexpansile C_3_F_8_ significantly reduced the time to resolution of acute corneal hydrops from symptom onset and initiation of therapy only in eyes with keratoconus but not in eyes with PMD or keratoglobus [23]. The main complication in this study was a reversible pupillary block seen in 16% of patients. 

Prolonged medical management in our patient, which included prednisolone acetate 1% and cyclopentalate eye drops as well as oral prednisone, was ultimately unsuccessful in ameliorating the chronic hydrops. Due to the inferior location of the Descemet membrane tear (from 4 to 7 o'clock) in our patient, the use of intracameral gas was not considered an appropriate option due to a high likelihood of failure and risk of developing pupillary block and glaucoma, and the inconvenience of requiring the patient to be supine position for several weeks. In addition, injection of gas through an already thinned peripheral cornea is not without potential complications. Therefore, a decision was made to perform a DSEK.

To our knowledge, this is the first report of a DSEK for an eye with keratoglobus and chronic hydrops secondary to a spontaneous Descemet membrane tear. Recently, Gorovoy et al. described their experience with Descemet stripping automated endothelial keratoplasty (DSAEK) for spontaneous Descemet membrane detachment in an Osteogenesis imperfecta patient with keratoconus and acute bullous keratopathy [24]. DSAEK was performed due to failed Descemet membrane reattachment with air bubbling, and seven months postoperatively the VA in the affected eye improved to 20/50 from HM preoperatively. 

Nonresolving hydrops secondary to spontaneous Descemet membrane tear complicating keratoglobus may result in permanent endothelial cell damage and scar formation. Our case demonstrates that these complications can be managed successfully with a DSEK. In addition, the complex structural abnormalities characterizing eyes with severe corneal ectasia may present significant surgical challenges potentially leading to unanticipated intraoperative complications such a cataract extraction in our patient.
